# Exposure to Traffic Density during Pregnancy and Birth Weight in a National Cohort, 2000–2017

**DOI:** 10.3390/ijerph19148611

**Published:** 2022-07-15

**Authors:** Marcelle Virginia Canto, Mònica Guxens, Rebeca Ramis

**Affiliations:** 1 Department of Preventive Medicine, Hospital Central de la Cruz Roja, 28003 Madrid, Spain; mcanto@salud.madrid.org; 2 Barcelona Institute for Global Health (ISGlobal), 08003 Barcelona, Spain; monica.guxens@isglobal.org; 3 Spanish Consortium for Research on Epidemiology and Public Health (CIBERESP), 28029 Madrid, Spain; 4 Department of Medicine and Live Sciences, Universitat Pompeu Fabra, 08002 Barcelona, Spain; 5 Department of Child and Adolescent Psychiatry/Psychology, Erasmus MC, University Medical Centre, 3015 GE Rotterdam, The Netherlands; 6 Chronic Diseases Department, National Centre for Epidemiology, Carlos III Institute of Health, 28029 Madrid, Spain

**Keywords:** low birthweight, pregnancy, traffic-related air pollution, road congestion

## Abstract

The variation on birth weight is associated with several outcomes early on in life and low birth weight (LBW) increases the risk of morbidity and mortality. Some environmental exposures during pregnancy, such as particulate matters and other traffic-related pollutants can have a significant effect on pregnant women and fetuses. The aim of this study is to estimate the effect of exposure to traffic density during pregnancy over birth weight in Spain, from 2000–2017. This was a retrospective, cross-sectional study using the information from Spain Birth Registry Statistics database. The traffic density was measured using the Annual average daily traffic. Multivariate linear regression models using birth weight and traffic density were performed, as well as a logistic regression model to estimated Odds ratios for LBW and GAM models to evaluate the non-linear effect. Our findings showed that increases in traffic density were associated with reduction of birth weight and increases of LBW risk. Moreover, exposure to high and very-high traffic-density during pregnancy were associated with reduction of birth weight and increase on LBW risk comparing with exposure to low number of cars trespassing the neighborhoods. The results of this study agree with previous literature and highlights the need of effective policies for reducing traffic density in residential neighborhoods of cities and towns.

## 1. Introduction

The World Health Organization (WHO) define children born with low birth weight (LBW), as a newborn who weigh less than 2500 g at birth [1]. The variation in birth weight is associated with several outcomes early on in life, such as neonatal mortality and morbidity, delayed growth retardation and poor cognitive and behavioral development, as well as chronic diseases displayed in adulthood, including diabetes mellitus, hypertension, and obesity [2]. One of the major goals in “A World Fit for Children”, a plan of the United Nations General Assembly Special Session on Children, is to reduce LBW incidence. It has been estimated that LBW babies are approximately 20 times more likely to die during the neonatal period than heavier babies due to poor health outcomes [1]. More than 20 million infants worldwide (15.5%) are born with LBW [1]. In 2015, Spain registered an 8.3% of newborns with LBW, a percentage that have been incrementing exponentially since 2000 [3].

Pollutants and contaminants such as particulate matter (PM), especially PM of 2.5 μm or less (PM_2.5_) and those particulates with 10 μm or less in diameter (PM_10_), sulfur dioxide (SO_2_), carbon monoxide (CO), nitrogen dioxide (NO_2_) and ozone (O_3_) are associated with traffic-related air pollution (TRAP) [4,5], what is one of the major sources of contamination in the world’s most populated and larger cities [6,7,8]. It has been estimated that about 100,000 of deaths per year worldwide could be linked to this exposure [9]. Also, their harmful effect on birth outcomes have been displayed in several studies [2,10,11,12], and it has been estimated that about 100,000 of deaths per year worldwide could be linked to this exposure [9]. Recent meta-analyses have highlighted the significant association between air pollution due to traffic and LBW [13,14,15] and more studies have shown how adverse birth effects were linked to maternal residential proximity to a mayor traffic density area, to heavy traffic lights, to bus stations, and to several ring and major roads [9,16,17,18,19,20]. To support this evidence, it has been shown that air pollution can lead to an increase in cell proliferation, organ development changes and modification on fetal metabolism, that can cause especial vulnerability and damage during the states of pregnancy and the neonatal period [21,22,23,24,25].

Measuring traffic air pollution could be approached by different strategies, such as direct air quality monitoring, modeling air pollution, or distance to road or traffic density [13,14,15]. The correlation between air quality and traffic flow [6] makes possible to accurately estimate air pollution exposure using traffic density [15]. The definition of traffic density is the number of vehicles that fill a lane of a roadway section at a point of time, expressed as vehicles per mile or kilometers [26] and it has been shown that as traffic volume increases in a road, pollutant emission increases as well [27,28].

Previous studies have been conducted in Spain and similar countries [20,29], nevertheless no previous study has used the whole national cohort of births for 18 years, meaning millions of birth records. This study, therefore, aimed to estimate the association of exposure to traffic density during pregnancy with birth weight using a population-birth cohort for the period 2000 to 2017.

## 2. Materials and Methods

### 2.1. Data

#### 2.1.1. Study Design and Population

We carried out a retrospective, cross-sectional study using the information from Spain Birth Registry Statistics database from the National Statistics Institute (INE) was used. The initial population was 5,071,963 birth records from all the regions of Spain, between year 2000 and 2017, from which only pregnancy with one single live fetus born between gestation weeks 37 and 42 were selected. Children with missing data on birth weight or unplausible birth weight were excluded, resulting in a final population of 4,934,810 birth records.

#### 2.1.2. Traffic Density

The annual average daily traffic (AADT) is a measure of the traffic density. It is expressed as number of vehicles per day and it is calculated as the total volume of transportation traffic of a road or highway for a year divided, by 365 days. AADT is a tool that is use to predict how active a road is and it is a useful instrument when assessing the effect of traffic density exposure.

For the present study, AADT was measured using the Navteq cartography combined with the traffic density information provided by the Spanish Ministry of Public Words and Transport [30]. Using the geographic coordinates of the mother’s home address as listed in the municipal registry and included in the birth record, it was possible to estimate the traffic density at the mother’s residence, during pregnancy [30]. Precisely, we computed the traffic density for streets and other roads in a buffer of 100 m around the mother’s residential address, to obtain the AADT in front of the house. To ensure anonymization all addresses were geocoded within a buffer of 30 m around the house.

#### 2.1.3. Outcomes

Outcome variables were birth weight (in grams) as a continuous variable and LBW as binary. The neonatal weight was collected using the birth records and registers with less than 2000 g or more than 4650 g were excluded, because we considerate those weights as little plausible birth weight for a term pregnancy. We created the binary variable LBW using the neonatal weight in grams and defined as low those babies that weighted less than 2500 g.

#### 2.1.4. Covariates

**Figure 1:**
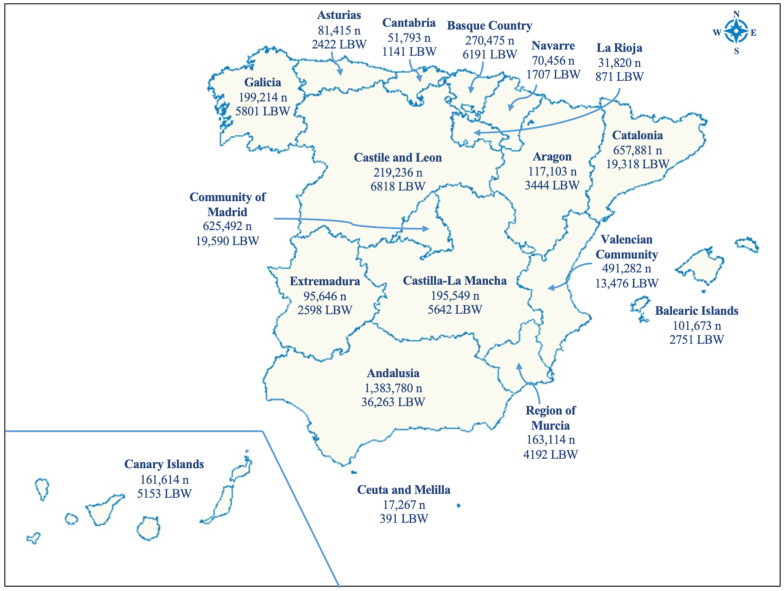
Map of the Spanish Regions with name, number of newborn (n) and number of newborns with LBW (LBW).

We obtain information related to the mother, father, gestation and neonatal factors at delivery from the birth records. We collected data on infant sex, birth year, mother’s age, father’s age, autonomous community (18 regions) (Figure 1), and size of municipality of the mother’s residence (equal or less than 10,000 inhabitants, 10,001 to 20,000 inhabitants, 20,001 to 50,000 inhabitants, 50,001 to 100,000 inhabitants, more than 100,000 inhabitants, and towns that are capital city of a province). Using the geographical coordinates of each mother’s home residence, declared in the birth records, we collected sociodemographic covariates, such as economic activity rate and socioeconomic status (SES), via census track official data of the INE. The economic activity rate, ranged from 20 to 100, is based on the Economic Active Population Survey, with reference to people that is actively working and the population aged 16 years and over [31]. SES, which ranged from 0.31 to 1.62, from worse to better socio-economic condition, is a key factor related to LBW and TRAP [32,33] and is based on the situation of the head of a family regarding their profession, occupation and activity [31].

### 2.2. Statistical Analysis

Before the analysis, we categorized the AADT variable into four groups using the quartiles of the roads traffic average (low, moderate, high and very high) and for the regression models; we use the lowest traffic density areas as the variable reference.

First, we performed a descriptive analysis of the sociodemographic variables, by percentages for categorical variables and means and standard deviations for continuous variables.

Then, in order to quantify the association between pregnancy’s exposure to traffic density and birth weight, we fitted multivariate linear regression models using birth weight as the dependent variable and traffic density as explanatory variable, in both forms as continuous and categorical. For LBW, we estimated Odds ratios (OR) and 95% confidence intervals (95% CI) using logistic regression. First, for both models we fitted an initial unadjusted model and then an adjusted model including gestation age, infant sex, birth year, maternal and paternal age, maternal autonomous community and size of municipality of residence, and maternal socio-economic condition and economic activity rate. We tested the potential modification effect of infant sex by including the interaction between traffic density and infant sex in the models. Finally, we fitted GAM models over the continuous variable to evaluate potential risk in a not linear fashion.

For all analyses and tests a *p*-value ≤ 0.05 was considered as the significant statistical level. The data was analyzed with the statistical program R 4.0.2.

## 3. Results

The study included 4,934,810 birth records and their neonatal and parental characteristics distribution are shown in Table 1. The gestational mean age was 39.4 weeks at birth, 51% were male infants and the majority of the birth records were registered between the periods of 2000–2004 and 2013–2017. The mean birth weight was 3294.4 g, and the percentage of the newborn with low birth weight was 2.8%. The highest and lowest incidence of LBW in Spain were 3.19% (Canary Island) and 2.20% (Cantabria) (Figure 1). The autonomous regions with the greatest number of births were Andalusia (28%), Catalonia (13.3%), Community of Madrid (12.7%) and Valencian Community (10%). There were two regions with the most population living in areas with more than 40% of very high traffic density in the roads near the home address of their birth records (Community of Madrid and Catalonia), and Galicia and Navarre were the regions with the less high and very high traffic densities. The maternal and paternal mean age was 32 and 34 years old, respectively, with the higher mean age in the group of very high traffic density near their home address. Woman living in regions with more than 100,000 inhabitants, with a higher socio-economic condition and higher activity rate, tended to be living around areas with a higher traffic density.

Table 2 shows the unadjusted and adjusted association between exposure to traffic density and birth weight. We use the lowest traffic density areas as reference, results showed an excess of weight loss with the increment of traffic density, with an average loss of 5 g in neonates born in areas with very high traffic density for the adjusted model. An increase in 100 cars passing by the mother’s home address during pregnancy was associated with a slightly decrease in birth weight (*p* < 0.01).

Detailed analyses of the unadjusted and adjusted association between each covariate and the birth weight of this analysis can be found in the annexes on the Supplementary Table S1. In brief, mothers that lived in regions with 50,001 to 100,000 inhabitants, who had higher socio-economic condition and lower economic activity rate had infant with a higher birth weight.

Table 3 shows the results for the logistic regression on LBW risk. Traffic density exposure during pregnancy was associated significantly with a risk of LBW. It could be seen that there was very small but positive effect at evaluating the traffic-density as a continuous variable in the adjusted model (OR = 1.005, *p* < 0.01), however, when the effect was evaluated by measuring the traffic density in quartiles, it shown more clearly that as the traffic density increments, the risk of LBW also increments exponentially, with a risk of 9% in the crude model and 8% in the adjusted model, in the group exposure to a very high traffic density compared to the group with the lower traffic density in the roads near their homes (*p* < 0.01).

Table 4 shows the odds ratio (ORs) and the respective 95% confidence intervals (95% CIs) of the covariates of study according to LBW between the newborn records. Results for the adjusted model showed a higher risk of LBW for mothers that lived in Castile and Leon, Community of Madrid, Canary Islands and Galicia (33%, 30% and 29% the last two regions), compared to babies born in Andalusia. On contrary, a lower risk was found for mothers living in Ceuta and Melilla, and Cantabria (17% and 9%, respectively). As well, mothers that resided on municipalities with more than 100,000 inhabitants, had 6% more risk of having newborns with LBW than those in the smaller towns. At evaluating the economic situation of the mother, higher socio-economic condition and lower economic activity rate during pregnancy were associated with decreased risk of LBW (36%, both conditions), however, the association wasn’t statistically significant for the maternal economic activity rate.

**Figure 2:**
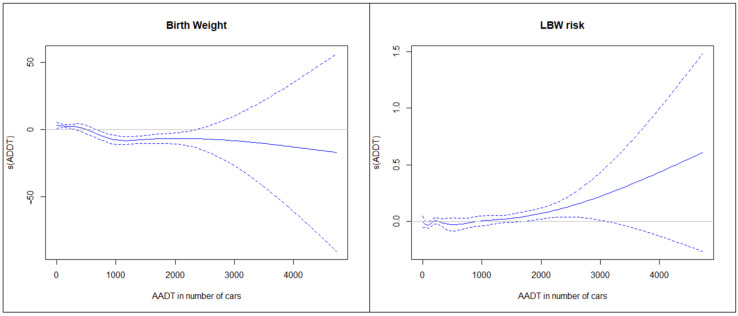
Result from the no linear models (GAM) for the birth weight (**left**) and LBW risk (**right**) related to Annual average daily traffic (AADT).

Finally, Figure 2 shows the evolution of both, birth weight and LBW risk, estimated by the GAM model. On the left-hand side graph, we can see how birth weight decrease with increasing traffic density and on the right-hand side we see an increase in LBW risk with the increase of traffic density.

## 4. Discussions

This study focused on the association between exposure to the traffic density over birth weight in Spain, considering traffic density close to the home address of pregnant women as proxy of traffic-related pollution exposure. Based on our results, an increasing number of cars trespassing near the mother’s home residence seems to be associated with a small reduction of weight at birth, nevertheless the effect over LBW risk is more obvious and exposure to high and very high traffic-density during pregnancy showed increased risk of LBW in comparison with exposure to low traffic density. Moreover, this study showed that the newborns with higher risk of LBW were also associated with mother’s residence in cities with more than 100,000 inhabitants.

As regard to the different proxies to the exposure, several studies investigated the effect of traffic-related air pollution using prediction models to estimate air pollution by calculating daily concentrations of PM, specifically PM_2.5_ and PM_10_, and other pollutants, such as SO_2_, CO, NO_2_ and O_3_, nearby the study groups [9,34,35,36]. Conversely, for the present study, we decided to use traffic density, calculated within circular buffers in meters on maternal home address based on AADT, due to the difficulty of having prediction models for the whole country during the 18 years of study, as well that traffic density can be considerate as a more valuable indicator of traffic-related pollution than streets length or the distance to roads [15].

A recent meta-analysis published in 2020, described positive associations between air pollution due to a higher traffic density and LBW, finding an OR of 1.013 (95% CI: 0.995, 1.031) for the risk of LBW with an increment of 5000 vehicles/day in traffic density, similarly to our results. In addition, they evaluated studies that treated traffic as a binary variable depending on the distance to the road. A positive effect between LBW and the distance to road was found as well, showing an OR of 1.044 (95% CI: 1.020, 1.068) when the cut-off point was 50 m and an OR of 1.023 (95% CI: 1.002, 1.042) with a distance of 100 m [15].

A Japanese study that evaluated the association between maternal residential proximity to major roads and adverse birth outcomes, determinate that living within 200 m from a major road increased the risk of preterm birth by 1.5 times and LBW by 1.2 times. It also pointed out that mothers with lower individual socio-economic position (SEP) showed higher ORs for term LBW (OR = 3.1, 95% CI: 1.2–8.2) compared with those with higher individual SEP [29]; comparable to our findings, were women with higher socio-economic conditions presents 36% less risk of LBW compared to women with very low socio-economic conditions. A similar study of 6438 singleton births in Spain (2001–2005), with distance to roads as proxy, found that living within the same distance (200 m) from a major road was associated with an increased risk of 46% of LBW, partly mediated by air pollution and heat exposure [20].

Another meta-analysis from 2018, which reviewed 15 studies of the association between air pollution exposure during different phases of pregnancy and fetal growth and anthropometric measurements at birth. Described a negative associated with Maternal PM_2_._5_ exposure during the entire pregnancy and head circumference at birth, as well as an association between maternal NO_2_ exposure and shorter length at birth [14]. However, Spanish official birth records do not included information of anthropometric measurements more that weight; therefore, we could not evaluate such a variable.

Previous studies showed that inhalation of pollutants during pregnancy, in particular those that are associated with traffic contamination, is associated with systemic inflammation, oxidative stress-induced DNA damage and, blood coagulability and viscosity alterations. All these can affect the placenta or the embryo directly, leading to LBW and others adverse birth outcomes [21,37,38]. A Spanish study with hundreds of pregnant women exposed to ambient NO_2_, suggested an association between maternal exposure to outdoor air pollution and birth outcomes. Its results showed an increased odd of being born small for gestational age (increased by 37% when ambient NO_2_ levels increased 10 μg/m^3^ during the second trimester) and being born at risk of lower length (reduction of −0.27 cm) or weight (reduction of −40.3 g) with a NO_2_ exposure above 40 μg/m^3^ during the first trimester [11].

In 2016, a study about the association between TRAP and adverse pregnancy outcomes was conducted in Canada by mapping the NO_2_ and PM_2_._5_ surfaces near the mother’s postal code during pregnancy. The authors estimated that LBW and term birth weight was strongly associated with both pollutants (reduction of 16.2 g, 95% CI 13.6–18.8 g per 20 ppb NO_2_) [39], similar to our finding, considering that a higher traffic density translates to a higher concentration of this specific pollutants in a neighborhood [11].

One study based in Brazil presented an interaction between infant sex and the exposure of pregnant women to air pollutants. This study suggested that sex of the newborn played a paper in the association of air pollutants exposure during pregnancy and LBW. Specially, exposure of ozone in the period of 30 and 90-day window prior to the birth, showed that female newborns presented a bigger susceptibility (OR = 1.58 and 1.59, respectively) to maternal exposure to air pollutants than male newborns. Conversely, a different study claimed to find a contrary result, suggesting that male fetuses are more sensitive to air pollution exposure, especially to PM_2.5_ [40]. Nevertheless, our study did not show the interaction between infant sex and exposure to traffic density during pregnancy in association with LBW risk.

As usual, this study has some limitations to mention. A main one is the no inclusion of data on previous maternal health history, substance use or complications or diseases during pregnancy that may affect the newborn’s weight. Other limitation is the use of the maternal home address as listed in the municipal registry at the moment of birth as location point of exposure estimation. Pregnant women could be out of home working or somewhere else and we did not include those other locations in the study that can led to inexact results if people’s mobility is not considered as stated in some studies [41,42]. We used traffic density as a proxy of traffic-related pollution instead of quantifying the exposure of each ambient air pollutant traffic-related, which is as well a limitation of our study. Nevertheless, the large sample size of this study, close to 5 million records, and inclusion of all Spanish regions during a period of 18 years, mitigate the potential bias due to the lack of information and increase the power of the resulting estimations.

In addition to these limitation, we much point out that the traffic density levels have improved in the Spanish main cities during the last few years, mostly due to changes related to traffic implemented policies and increased use of environmentally-friendly cars, as well as growing network of renting public electric cars, bicycle and skateboard around the center of major cities in Spain [43,44,45,46]. All these changes have made less probable to reach the maximum levels exposure showed in our study for future years. Also, a major event like COVID-19 pandemic that occurred posterior to our study period, has affected pollution emissions in a positive manner [47,48,49]. However, this improvement was probably not highly significant in long terms due to other modifications in daily conducts after returning to normality.

It is crucial to reduce TRAP and improve air quality by adopting and promoting effective policies to reduce air pollution emissions, as well as to increase awareness of the consequence of this exposure, especially to the population of reproductive age that live nearby high pollutant areas. Several initiatives have been taken place internationally, as well as at a European and national level related with this matter. One major key initiative was the creation of National Air Pollution Control Programme (NAPCP), approved in 2019 by the Council of Ministers [50]. The NAPCP includes 50 measures grouped into 8 sectoral packages, and estimated that by applying these measures, reductions of pollutants are ensured in 2030 by the following: 92% for SO_2_, 66% for NO_x_, 30% for non-methane volatile organic compounds, 21% for ammonia and 50% for PM_2.5_ [51].

## 5. Conclusions

The results of this study agree with previous literature and finds association between exposure to high traffic density and LBW risk. This result highlights the need of effective policies for reducing traffic density in residential neighborhoods of cities and towns.

## Figures and Tables

**Table 1:** Overall characteristics based on traffic and by quartile of traffic-density, 2000–2017 (*n* = 4,934,810).

		Traffic Density at Home Address			
Characteristics	Overall	Low Traffic Density	Moderate Traffic Density	High Traffic Density	Very High Traffic Density
**Newborn variables**					
Gestation age at birth, mean (weeks)	39.43	39.44	39.43	39.43	39.40
Birth weight, mean (grams)	3294.40	3297.40	3298.17	3296.75	3285.29
Low birth weight (%)	2.79	24.25	24.42	25.07	26.26
Infant sex (%)					
Male	51.33	24.99	25.02	24.97	25.02
Female	48.67	25.01	24.98	25.03	24.98
Birth year (%)					
2000–2004	26.90	23.67	24.30	25.98	26.04
2005–2008	23.40	24.59	25.04	25.44	24.92
2009–2012	22.99	25.99	25.53	24.34	24.15
2013–2017	26.71	25.84	25.22	24.19	24.75
**Parental variables**					
Maternal age (%)					
<20	3.13	25.16	23.99	29.57	21.28
20–34	67.81	25.52	24.80	25.40	24.27
35–40	26.24	23.81	25.61	23.59	27.00
>40	2.81	23.38	25.17	23.45	27.99
Maternal age, mean (years)	31.53	31.36	31.64	31.22	31.92
Paternal age, mean (years)	33.97	34.01	34.06	33.70	34.13
Maternal autonomous community of residence (%)					
Andalusia	28.04	25.98	24.18	28.29	21.55
Aragon	2.37	39.55	38.45	18.71	3.29
Asturias	1.65	32.93	44.27	19.25	3.55
Balearic Islands	2.06	30.64	39.35	28.44	1.58
Basque Country	5.48	24.25	30.20	16.25	29.30
Canary Islands	3.27	19.36	25.87	33.29	21.48
Cantabria	1.05	31.94	41.17	23.37	3.51
Castile and Leon	4.44	38.62	34.74	20.71	5.94
Castilla-La Mancha	3.96	47.84	24.65	22.55	4.96
Catalonia	13.33	15.39	15.63	21.67	47.30
Ceuta and Melilla	0.35	18.63	38.95	37.94	4.49
Community of Madrid	12.68	12.86	15.40	21.36	50.37
Extremadura	1.94	43.58	26.26	24.59	5.57
Galicia	4.04	40.07	42.53	14.77	2.63
La Rioja	0.64	40.40	41.52	15.79	2.29
Navarre	1.43	45.27	36.78	16.14	1.80
Region of Murcia	3.31	23.04	28.16	47.53	1.27
Valencian Community	9.96	18.21	21.99	29.87	29.93
Maternal size of municipality and capital of residence (%)					
Equal or less than 10,000 inhabitants	17.32	70.28	11.83	12.13	5.76
10,001–20,000 inhabitants	11.64	30.64	27.16	29.83	12.37
20,001–50,000 inhabitants	16.66	16.32	30.51	33.47	19.70
50,001–100,000 inhabitants	10.89	11.72	30.61	31.78	25.89
More than 100,000 inhabitants	10.57	9.23	23.11	23.41	44.25
Capital of the province	32.92	13.02	27.13	24.05	35.80
Maternal socio-economic condition (%)					
0.31–0.57	0.47	20.23	26.07	33.12	20.58
0.58–0.83	14.17	38.19	21.42	28.09	12.31
0.84–1.09	60.55	23.88	26.51	25.96	23.65
1.10–1.35	24.18	20.67	23.65	20.79	34.89
1.36–1.62	0.63	5.46	11.91	18.72	63.91
Maternal socio-economic condition, mean	0.99	0.96	1.00	0.98	1.04
Maternal economic activity rate (%)					
20–40	0.00	95.18	4.82	0.00	0.00
41–60	0.66	43.62	24.88	22.44	9.06
61–80	78.54	25.84	25.53	25.60	23.04
81–100	20.80	21.24	23.02	22.83	32.92
Maternal economic activity rate, mean	75.42	74.49	75.22	75.10	76.86

**Table 2:** Effect of traffic density of maternal home address on mean weight at birth during 2000–2017 in Spain.

Characteristics	Unadjusted Association		Adjusted Association ^a^	
	Grams (95% CI)	*p*-Value	Grams (95% CI)	*p*-Value
**Traffic-density, per 100 cars**	−0.87 (−0.94, −0.81)	<0.01	−0.33 (−0.40, −0.26)	<0.01
**Traffic-density, per quartiles**				
Low traffic density	Ref.		Ref.	
Moderate traffic density	0.77 (−0.29, 1.83)	0.10	−1.65 (−2.74, −0.55)	<0.01
High traffic density	−0.65 (−1.71, 0.41)	0.10	−2.02 (−3.12, −0.96)	<0.01
Very high traffic density	−12.11 (−13.17, −11.04)	<0.01	−5.10 (−6.30, −3.89)	<0.01

95% CI: 95% confidence interval. Ref.: Reference category. ^a^: Adjusted changes in mean birth weight for parity, maternal and paternal characteristic, including gestational age, infant sex, year of birth, maternal and paternal age, maternal autonomous community and size of municipality and capital of residence, maternal socio-economic condition and economic activity rate.

**Table 3:** Crude and adjusted Odds ratio for low birth weight (<2500 g) and traffic density during 2000–2017 in Spain.

Characteristics	Crude OR		Adjusted OR ^a^	
	OR (CI 95%)	*p*-Value	OR (CI 95%)	*p*-Value
**Traffic-density, per 100 cars**	1.006 (1.005, 1.007)	<0.01	1.005 (1.004, 1.006)	<0.01
**Traffic-density, per quartiles**				
Low traffic density	Ref.		Ref.	
Moderate traffic density	1.01 (0.99, 1.02)	0.10	1.01 (0.99, 1.03)	0.10
High traffic density	1.04 (1.02, 1.05)	<0.01	1.04 (1.02, 1.05)	<0.01
Very high traffic density	1.09 (1.07, 1.10)	<0.01	1.08 (1.06, 1.10)	<0.01

CI 95%: 95% confidence interval. Ref.: Reference category. ^a^: Adjusted low birth weight outcome associated with parity, maternal and paternal characteristics, including gestational age, infant sex, year of birth, maternal and paternal age, maternal autonomous community and size of municipality and capital of residence, maternal socio-economic condition and economic activity rate.

**Table 4:** Adjusted Odds ratio for low birth weight (<2500 g) during 2000–2017 in Spain.

Characteristics	Crude OR		Adjusted OR ^a^	
	OR (95% CI)	*p*-Value	OR (95% CI)	*p*-Value
**Gestation age at birth (weeks)**	0.47 (0.46, 0.47)	<0.01	0.46 (0.46, 0.46)	<0.01
**Infant Sex**				
Male	Ref.		Ref.	
Female	1.55 (1.53, 1.56)	<0.01	1.62 (1.61, 1.64)	<0.01
**Birth year**	1.00 (1.00, 1.01)	<0.01	1.01 (1.01, 1.01)	<0.01
**Maternal age (years)**	0.99 (0.99, 1.00)	<0.01	0.99 (0.99, 0.99)	<0.01
**Paternal age (years)**	0.99 (0.99, 0.99)	<0.01	1.00 (0.99, 1.00)	<0.01
**Maternal autonomous community of residence**				
Andalusia	Ref.		Ref.	
Aragon	1.13 (1.09, 1.17)	<0.01	1.28 (1.23, 1.33)	<0.01
Asturias	1.14 (1.09, 1.19)	<0.01	1.23 (1.18, 1.29)	<0.01
Balearic Islands	1.03 (0.99, 1.07)	0.10	1.08 (1.04, 1.13)	<0.01
Basque Country	0.87 (0.85, 0.89)	<0.01	0.97 (0.94, 1.00)	0.01
Canary Islands	1.22 (1.19, 1.26)	<0.01	1.29 (1.25, 1.33)	<0.01
Cantabria	0.84 (0.79, 0.89)	<0.01	0.91 (0.86, 0.97)	<0.01
Castile and Leon	1.19 (1.16, 1.22)	<0.01	1.33 (1.29, 1.37)	<0.01
Castilla-La Mancha	1.10 (1.07, 1.14)	<0.01	1.10 (1.06, 1.13)	<0.01
Catalonia	1.12 (1.10, 1.14)	<0.01	1.24 (1.22, 1.27)	<0.01
Ceuta and Melilla	0.86 (0.78, 0.95)	<0.01	0.83 (0.75, 0.92)	<0.01
Community of Madrid	1.20 (1.18, 1.22)	<0.01	1.30 (1.27, 1.33)	<0.01
Extremadura	1.04 (1.00, 1.08)	0.05	1.08 (1.04, 1.13)	<0.01
Galicia	1.11 (1.08, 1.15)	<0.01	1.29 (1.25, 1.32)	<0.01
La Rioja	1.05 (0.98, 1.12)	0.10	1.13 (1.05, 1.21)	<0.01
Navarre	0.92 (0.88, 0.97)	<0.01	1.02 (0.97, 1.08)	0.10
Region of Murcia	0.98 (0.95, 1.01)	0.10	0.99 (0.96, 1.03)	0.10
Valencian Community	1.05 (1.03, 1.07)	<0.01	1.04 (1.02, 1.06)	<0.01
**Maternal size of municipality and capital of residence**				
Equal or less than 10,000 inhabitants	Ref.		Ref.	
10,001–20,000 inhabitants	1.01 (0.99, 1.03)	0.10	1.02 (1.00, 1.05)	0.01
20,001–50,000 inhabitants	1.01 (0.99, 1.03)	0.10	1.00 (0.98, 1.02)	0.10
50,001–100,000 inhabitants	1.04 (1.02, 1.06)	<0.01	1.02 (1.00, 1.05)	0.05
More than 100,000 inhabitants	1.13 (1.10, 1.15)	<0.01	1.06 (1.03, 1.08)	<0.01
Capital of the province	1.05 (1.04, 1.07)	<0.01	1.03 (1.02, 1.05)	<0.01
**Maternal socio-economic condition**				
0.31–0.57	Ref.		Ref.	
0.58–0.83	0.93 (0.86, 1.00)	0.01	0.96 (0.89, 1.04)	0.10
0.84–1.09	0.89 (0.83, 0.96)	<0.01	0.84 (0.77, 0.91)	<0.01
1.10–1.35	0.80 (0.74, 0.86)	<0.01	0.69 (0.64, 0.75)	<0.01
1.36–1.62	0.81 (0.73, 0.89)	<0.01	0.64 (0.58, 0.72)	<0.01
**Maternal economic activity rate**				
20–40	Ref.		Ref.	
41–60	0.85 (0.27, 2.70)	0.10	0.64 (0.20, 2.07)	0.10
61–80	0.77 (0.24, 2.43)	0.10	0.65 (0.20, 2.11)	0.10
81–100	0.75 (0.24, 2.38)	0.10	0.67 (0.21, 2.18)	0.10

95% CI: 95% confidence interval. Ref.: Reference category. ^a^: Adjusted changes in low birthweight outcome for each variable, including traffic density.

## Data Availability

The data presented in this study are available on request from the corresponding author.

## Supplementary Materials

The following supporting information can be downloaded at: https://www.mdpi.com/article/10.3390/ijerph19148611/s1, Table S1: Effect of sociodemographic characteristics and traffic density on mean weight at birth during 2000–2017 in Spain.
