# Supplementary material for: Exposure to Traffic Density during Pregnancy and Birth Weight in a National Cohort, 2000–2017
Source: Int J Environ Res Public Health. 2022 Jul 15;19(14):8611. doi: 10.3390/ijerph19148611 (PMC9318762; doi:10.3390/ijerph19148611)
Supplement: Supplementary file 1 [file ijerph-19-08611-s001.zip › ijerph-1764620-supplementary.pdf]

Supplementary

Supplementary Table S1. Effect of sociodemographic characteristics and traffic density on mean weight at birth during 2000-2017 in Spain**.**

Characteristics	Unadjusted association		Adjusted association[a]	
	Grams (CI 95%)	p-value	Grams (CI 95%)	p-value
**Gestation age at birth (weeks)**	118.26 (117.96, 118.55)	< 0.01	119.50 (119.21, 119.79)	< 0.01
**Infant Sex**				
Male	Ref.		Ref.	
Female	−124.79 (−125.54, −124.05)	< 0.01	−129.4 (−130.10, −128.70)	< 0.01
**Birth year**	1.54 (1.47, 1.61)	< 0.01	0.67 (0.60, 0.74)	< 0.01
**Maternal age (years)**	1.68 (1.61, 1.75)	< 0.01	1.71 (1.62, 1.81)	< 0.01
**Paternal age (years)**	2.33 (2.26, 2.39)	< 0.01	2.10 (2.01, 2.18)	< 0.01
**Maternal autonomous community of residence**				
Andalusia	Ref.		Ref.	
Aragon	−60.68 (−63.21, −58.14)	< 0.01	−69.71 (−72.14, −67.28)	< 0.01
Asturias	−54.99 (−58.00, −51.99)	< 0.01	−62.36 (−65.22, −59.50)	< 0.01
Balearic Islands	−28.09 (−30.79, −25.38)	< 0.01	−22.47 (−25.06 −19.88)	< 0.01
Basque Country	−7.57 (−9.32, −5.82)	< 0.01	−17.30 (−19.00, −15.60)	< 0.01
Canary Islands	−31.15 (−33.34, −28.96)	< 0.01	−35.56 (−37.63, −33.49)	< 0.01
Cantabria	−25.30 (−29.03, −21.57)	< 0.01	−30.87 (−34.38, −27.36)	< 0.01
Castile and Leon	−76.07 (−77.98, −74.15)	< 0.01	−83.98 (−85.83, −82.13)	< 0.01
Castilla-La Mancha	−72.54 (−74.55, −70.53)	< 0.01	−64.44 (−66.35, −62.53)	< 0.01
Catalonia	−45.71 (−46.96, −44.47)	< 0.01	−52.17 (−53.47, −50.87)	< 0.01
Ceuta and Melilla	44.68 (38.30, 51.06)	< 0.01	45.80 (39.68, 51.92)	< 0.01
Community of Madrid	−80.69 (−81.96, −79.43)	< 0.01	−84.59 (−85.90, −83.28)	< 0.01
Extremadura	−48.46 (−51.24, −45.67)	< 0.01	−48.69 (−51.30, −46.08)	< 0.01
Galicia	−29.20 (−31.20, −27.20)	< 0.01	−47.38 (−49.31, −45.45)	< 0.01
La Rioja	−48.28 (−53.01, −43.56)	< 0.01	−41.04 (−45.48, −36.60)	< 0.01
Navarre	−13.56 (−16.78, −10.35)	< 0.01	−17.24 (−20.30, −14.18)	< 0.01
Region of Murcia	−21.47 (−23.65, −19.29)	< 0.01	−28.13 (−30.20, −26.06)	< 0.01
Valencian Community	−59.29 (−60.67, −57.91)	< 0.01	−50.28 (−51.64, −48.92)	< 0.01
**Maternal size of municipality and capital of residence**				
Equal or less than 10,000 inhabitants	Ref.		Ref.	
10,001-20,000 inhabitants	9.55 (8.12, 10.97)	< 0.01	3.01 (1.63, 4.40)	< 0.01
20,001-50,000 inhabitants	5.51 (4.22, 6.80)	< 0.01	1.96 (0.64, 3.28)	< 0.01
50,001-100,000 inhabitants	8.62 (7.17, 10.08)	< 0.01	4.86 (3.37, 6.34)	< 0.01
More than 100,000 inhabitants	−8.67 (−10.14, −7.20)	< 0.01	−3.70 (−5.24, −2.17)	< 0.01
Capital of the province	−4.68 (−5.80, −3.57)	< 0.01	−3.17 (−4.37, −1.97)	< 0.01
**Maternal socio-economic condition**				
0.31-0.57	Ref.		Ref.	
0.58-0.83	−5.92 (−11.53, −0.32)	0.01	−7.44 (−12.85, −2.02)	< 0.01
0.84-1.09	−14.03 (−19.56, −8.49)	< 0.01	12.11 (6.71, 17.51)	< 0.01
1.10-1.35	−12.32 (−17.89, −6.76)	< 0.01	26.78 (21.31, 32.25)	< 0.01
1.36-1.62	−27.21 (−34.47, −19.95)	< 0.01	33.14 (26.11, 40.17)	< 0.01
**Maternal economic activity rate**				
20-40	Ref.		Ref.	
41-60	53.25 (−38.56, 145.06)	0.10	78.93 (−7.55, 165.41)	0.05
61-80	58.00 (−33.69, 149.69)	0.10	69.58 (−16.80, 155.96)	0.10
81-100	52.71 (−38.98, 144.40)	0.10	66.27 (−20.11, 152.65)	0.10

CI 95%: 95% confidence interval. Ref.: Reference category.

[a]Adjusted changes in birth weight for each variable and traffic density.
